# Exon resequencing of H3K9 methyltransferase complex genes, *EHMT1*, *EHTM2* and *WIZ*, in Japanese autism subjects

**DOI:** 10.1186/2040-2392-5-49

**Published:** 2014-10-06

**Authors:** Shabeesh Balan, Yoshimi Iwayama, Motoko Maekawa, Tomoko Toyota, Tetsuo Ohnishi, Manabu Toyoshima, Chie Shimamoto, Kayoko Esaki, Kazuo Yamada, Yasuhide Iwata, Katsuaki Suzuki, Masayuki Ide, Motonori Ota, Satoshi Fukuchi, Masatsugu Tsujii, Norio Mori, Yoichi Shinkai, Takeo Yoshikawa

**Affiliations:** Laboratory for Molecular Psychiatry, RIKEN Brain Science Institute, 2-1 Hirosawa, Wako, Saitama, 351-0198 Japan; Department of Psychiatry and Neurology, Hamamatsu University School of Medicine, Hamamatsu, Shizuoka, Japan; Department of Psychiatry, Division of Clinical Medicine, University of Tsukuba, Tsukuba, Ibaraki, Japan; Graduate School of Information Science, Nagoya University, Nagoya, Aichi, Japan; Faculty of Engineering, Maebashi Institute of Technology, Maebashi, Japan; Faculty of Sociology, Chukyo University, Chukyo, Aichi, Japan; Research Center for Child Mental Development, Hamamatsu University School of Medicine, Hamamatsu, Shizuoka, Japan; Cellular Memory Laboratory, RIKEN, Wako, Saitama, Japan; CREST (Core Research for Evolutionary Science and Technology), Japan Science and Technology Agency, Kawaguchi, Saitama, Japan

**Keywords:** Autism, Rare variant, GLP, G9a, Wiz, Histone methyltransferase, H3K9

## Abstract

**Background:**

Histone H3 methylation at lysine 9 (H3K9) is a conserved epigenetic signal, mediating heterochromatin formation by trimethylation, and transcriptional silencing by dimethylation. Defective GLP (*Ehmt1*) and G9a (*Ehmt2*) histone lysine methyltransferases, involved in mono and dimethylation of H3K9, confer autistic phenotypes and behavioral abnormalities in animal models. Moreover, *EHMT1* loss of function results in Kleefstra syndrome, characterized by severe intellectual disability, developmental delays and psychiatric disorders. We examined the possible role of histone methyltransferases in the etiology of autism spectrum disorders (ASD) and suggest that rare functional variants in these genes that regulate H3K9 methylation may be associated with ASD.

**Methods:**

Since G9a-GLP-Wiz forms a heteromeric methyltransferase complex, all the protein-coding regions and exon/intron boundaries of *EHMT1, EHMT2* and *WIZ* were sequenced in Japanese ASD subjects. The detected variants were prioritized based on novelty and functionality. The expression levels of these genes were tested in blood cells and postmortem brain samples from ASD and control subjects. Expression of *EHMT1* and *EHMT2* isoforms were determined by digital PCR.

**Results:**

We identified six nonsynonymous variants: three in *EHMT1*, two in *EHMT2* and one in *WIZ*. Two variants, the *EHMT1* ankyrin repeat domain (Lys968Arg) and *EHMT2* SET domain (Thr961Ile) variants were present exclusively in cases, but showed no statistically significant association with ASD. The *EHMT2* transcript expression was significantly elevated in the peripheral blood cells of ASD when compared with control samples; but not for *EHMT1* and *WIZ*. Gene expression levels of *EHMT1, EHMT2* and *WIZ* in Brodmann area (BA) 9, BA21, BA40 and the dorsal raphe nucleus (DoRN) regions from postmortem brain samples showed no significant changes between ASD and control subjects. Nor did expression levels of *EHMT1* and *EHMT2* isoforms in the prefrontal cortex differ significantly between ASD and control groups.

**Conclusions:**

We identified two novel rare missense variants in the *EHMT1* and *EHMT2* genes of ASD patients. We surmise that these variants alone may not be sufficient to exert a significant effect on ASD pathogenesis. The elevated expression of *EHMT2* in the peripheral blood cells may support the notion of a restrictive chromatin state in ASD, similar to schizophrenia.

**Electronic supplementary material:**

The online version of this article (doi:10.1186/2040-2392-5-49) contains supplementary material, which is available to authorized users.

## Background

Autism spectrum disorders (ASD), characterized by defects in social reciprocity, impairment in communication and restricted and repetitive stereotyped behavioral patterns, are the most prevalent childhood neurodevelopmental disorders. They affect all racial, ethnic and socioeconomic groups equally, with a worldwide prevalence of approximately 0.6% [1, 2]. The genetic influences in the etiology of ASD have been demonstrated in family and twin studies [3, 4], along with discoveries of common and rare genetic variants and pronounced chromosomal abnormalities [5]. Recently, *de novo* rare variants with a large effect size were found to increase ASD susceptibility [6, 7]. However, generation of the ASD phenotype requires interaction between environmental factors, and inherited and *de novo* genetic variants [8]. Furthermore, the pivotal role of epigenetic regulatory mechanisms involved in the pathogenesis of Rett syndrome, fragile X syndrome and the identification of ASD-associated genetic defects in imprinted regions lends strength to the hypothesis that epigenetic factors are causative in ASD etiology [9].

Epigenetic mechanisms involving post translational modification of histone lysine methylation influence numerous biological processes, including transcription, replication and chromosome maintenance, all of which are tightly regulated by methyltransferases and demethylases [10]. Among them, methylation of lysine 9 in histone H3 (H3K9), marks a conserved epigenetic signal; by heterochromatin formation through trimethylation (H3K9me3) and transcriptional silencing through dimethylation (H3K9me2) [11]. The formation of H3K9me1 and H3K9me2 are mediated by a Suv39h subgroup of histone methyl transferases, namely G9a/KMT1C and GLP/KMT1D, both having Su(var)3-9-Enhancer of zeste-Trithorax (SET) domain, through which they form homomeric and heteromeric complexes [12]. The G9a-GLP heteromeric complex is known to interact with Wiz, a multi-zinc finger-containing molecule, resulting in a stable and dominant intracellular heteromeric methyltransferase complex [13].

Regulation of H3K9 methylation has a powerful impact on neurological function and disease, as exemplified in Kleefstra syndrome. This disease is characterized by severe intellectual disability, developmental delay and psychiatric disorders, and is the result of a 9q34 subtelomeric deletion and loss-of-function mutations in *EHMT1*[14, 15]. In *Ehmt1* heterozygous knockout mice, the typical autistic-like features including reduced exploration, increased anxiety, altered social behavior, deficits in fear extinction, and learning and object recognition (novel and spatial) are observed [16, 17]. Furthermore, the lack of postnatal and neuron-specific GLP/G9a expression in mouse models dysregulates neuronal transcriptional, resulting in behavioral abnormalities, such as impaired learning, motivation and environmental adaptation [18].

Therefore, the autistic-like features and behavioral abnormalities precipitated by defects in histone methyltransferases provide a powerful case for examining their involvement in ASD pathogenesis. We put forward that rare functional variants in these genes may be associated with ASD. Since G9a-GLP-Wiz forms a stable and dominant heteromeric methyltransferase complex in H3K9 methylation, we set out to resequence the *EHMT1, EHMT2* and *WIZ* genes coding for GLP, G9a and WIZ, respectively, in Japanese ASD case and control samples.

## Methods

### Subjects

A cohort of 315 patients of Japanese descent, with autism (262 males and 53 females, mean age ± SD =12.09 ± 5.72 years), comprising 293 independent subjects and affected siblings, were recruited for the resequencing studies. The diagnosis of autism was made using the *Diagnostic and Statistical Manual, Fourth Edition, Text Revision* (DSM-IV-TR: American Psychiatric Association, 2000) criteria. The Autism Diagnostic Interview-Revised (ADI-R) [19] was conducted by experienced child psychiatrists who are licensed to use the Japanese version of the ADI-R. Participants with comorbid psychiatric illnesses were excluded by means of the Structured Clinical Interview for DSM-IV (SCID) [20]. Control subjects (n =1,140, 440 males and 700 females, mean age ± SD =44.10 ± 13.63 years) devoid of any past or present psychiatric disorders were recruited from hospital staff and company employees. Samples were also collected from available parents of subjects who harbored novel mutations, in order to determine whether these mutations were *de novo*. All participants were provided with, and received a full explanation of study protocols and objectives, before giving informed, written consent to participate in the study. For patients under the age of 16 years, written informed consent was also obtained from their parents. The study was approved by the Ethics Committees of RIKEN and Hamamatsu University School of Medicine, and conducted according to the principles expressed in the Declaration of Helsinki. DNA was extracted from whole blood according to a standard protocol.

A subset of subjects, 52 ASD (43 males and 9 females, mean age ± SD =11.98 ± 2.43) and 32 normal controls (26 males and 6 females, mean age ± SD =12.31 ± 2.01), was selected to analyze transcript expression levels in peripheral blood cells from the cohort whose DNA was resequenced for the candidate genes. Postmortem brain tissues from ASD and age-matched control samples were obtained from the National Institute of Child Health and Human Development (NICHD) Brain and Tissue Bank, University of Maryland School of Medicine (http://medschool.umaryland.edu/btbank/), for gene expression analysis (Additional file 1: Table S1). Frozen tissue samples from BA09 (ASD; n =10, control; n =10), BA21 (ASD; n =14, control; n =14), BA40 (ASD; n =14, control; n =13) and DoRN regions (ASD; n =8, control; n =8) were used in this study. Total RNA from peripheral blood cells and brain tissues was extracted using a miRNAeasy Mini kit (QIAGEN GmbH, Hilden, Germany) and single stranded cDNA was synthesized using a SuperScript VILO cDNA synthesis kit (Life Technologies Co., Carlsbad, CA, USA), according to the manufacturers’ instructions.

### Resequencing and variant analysis

Protein-coding regions and exon/intron boundaries of *EHMT1, EHMT2* and *WIZ* were screened for variants in ASD case samples by direct sequencing of PCR products, using the BigDye Terminator v3.1 cycle Sequencing Kit (Applied Biosystems (ABI), Foster City, CA, USA), and analyzed on an ABI3730 Genetic Analyzer (ABI), using standard protocols. The primers used for amplification and PCR conditions are listed in Additional file 2: Table S2. The sequences were aligned to the respective reference sequences (*EHMT1* isoform 1: RefSeq NM_024757.4, Isoform 2: RefSeq NM_001145527.1, *EHMT2* isoform a: RefSeq NM_006709.3, isoform b: RefSeq NM_25256.5, and *WIZ*: RefSeq NM_021241.2) and variants were detected using Sequencher software (Gene Codes Corporation, Ann Arbor, MI, USA). For the heterozygous variant calls in Sequencher, the height of the secondary peak was set at 35% of the primary peak and all variants were confirmed by bidirectional sequencing of the sample.

**Figure 1:**
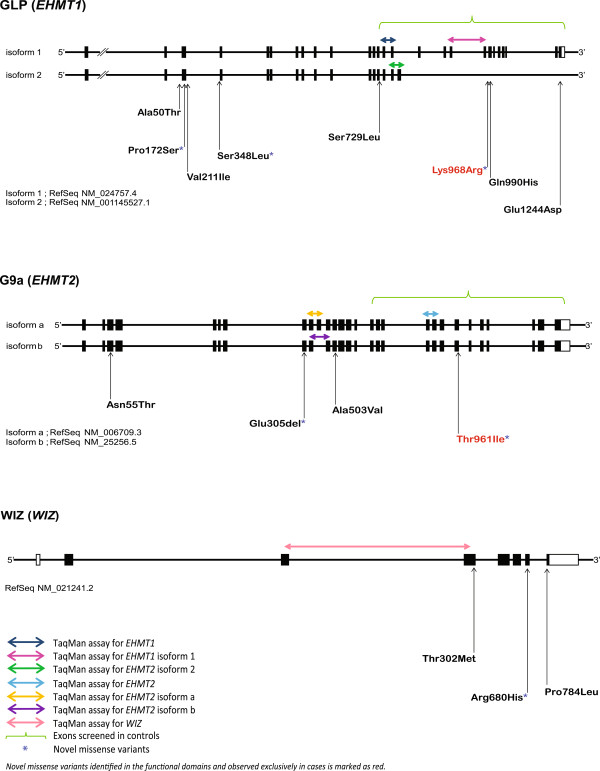
**Genomic structures of** ***EHMT1*** **,** ***EHMT2*** **and** ***WIZ*** **genes screened in Japanese autism spectrum disorder (ASD) subjects, and identified missense variants.** Black boxes denote coding exons and white boxes denote non-coding exons.

Variants were prioritized based on whether they were, (i) located in an important functional domain of the protein, (ii) deemed to be functional, such as a frame shift, stop gain or nonsynonymous mutation, and (iii) novel, that is not documented in the NCBI dbSNP database (Build 137) (http://www.ncbi.nlm.nih.gov/SNP/), the 1000 Genomes Project (http://www.1000genomes.org/), the Exome Variant Server of NHLBI GO Exome Sequencing Project (ESP6500SI-V2) (http://evs.gs.washington.edu/EVS/) or the Human Genetic Variation Database of Japanese genetic variation consortium (http://www.genome.med.kyoto-u.ac.jp/SnpDB). The potential functional consequences of variants were evaluated *in silico,* using PolyPhen-2 (http://genetics.bwh.harvard.edu/pph2/), PROVEAN (http://provean.jcvi.org/index.php) and SIFT (http://sift.jcvi.org/). In the control samples, we screened only exons coding for functional domains of the candidate genes (Figure 1 and Additional file 3: Figure S1 (A)). Fisher’s exact test (two-tailed) was used to compare the differences in allele counts between ASD and control subjects, with statistical significance being defined as *P* <0.05.

### Gene expression analysis

Real-time quantitative RT-PCR analysis was conducted using standard procedures, in an ABI7900HT Fast Real-Time PCR System (ABI, Foster City, CA, USA). TaqMan probes and primers for *EHMT1, EHMT2* and *WIZ* and *GAPDH* (internal control) were chosen from TaqMan Gene Expression Assays (ABI, Foster City, CA, USA) (Figure 1 and Additional file 4: Table S3). All real-time quantitative RT-PCR reactions were performed in triplicate, based on the standard curve method. To check for isoform-specific expressional changes between ASD cases and controls (prefrontal cortex), digital PCR was performed using standard procedures for *EHMT1* (variant 1: NM_024757.4 and variant 2: NM_001145527.1) and *EHMT2* (isoform a: NM_006709.3 and isoform b: NM_025256.5) isoforms, using TaqMan Gene Expression Assays in a QuantStudio12K Flex Real-Time PCR System (Life Technologies Co., Carlsbad, CA, USA) (Figure 1 and Additional file 4: Table S3). Significant changes in target gene expression levels between the cases and controls were detected by Mann–Whitney *U*-test (two-tailed) and *P* values of <0.05 were considered statistically significant.

## Results

### Resequencing and genetic association analyses

**Figure 2:**
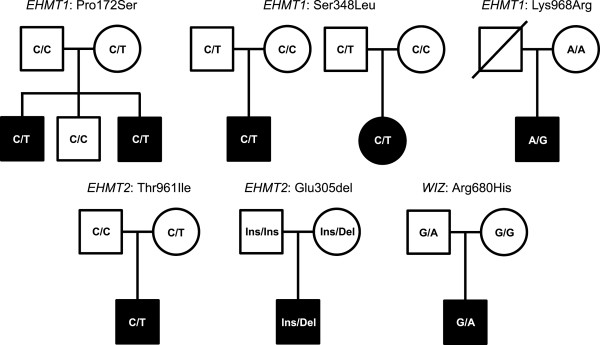
**Pedigree structures of autism spectrum disorder (ASD) families harboring novel missense variants in** ***EHMT1, EHMT2*** **and** ***WIZ*** **.** With the exception of Lys968Arg, none of the novel variants were *de novo*. For the Lys968Arg variant, genotype information of the father was not available.

Resequencing of the coding regions and exon/intron boundaries of the three genes, yielded several novel and previously reported variants in the ASD cohort, with varying minor allele frequencies (Additional file 5: Table S4). Filtering of variants based on functionality (nonsynonymous and frameshift) and novelty, revealed three nonsynonymous variants in *EHMT1*, two nonsynonymous variants in *EHMT2* and one nonsynonymous variant in *WIZ* (Table 1). All variants showed low minor allele frequencies (MAF <0.01) and were deemed to be inherited from the parents, although this could not be confirmed in cases bearing the *EHMT1* variant, Lys968Arg, due to a lack of parental samples for testing (Figure 2).

**Table 1:** **Novel missense variants identified in** ***EHMT1*** **,** ***EHMT2*** **and** ***WIZ*** **genes from autism spectrum disorders (ASD) cases and controls**

Gene	Chromosome position	Exon	cDNA position	Amino acid change	Protein domain	Autism count	Control count*	PolyPhen2	Provean	SIFT
*EHMT1*	9,140611506,C,T	Exon3	c.514C > T	p.Pro172Ser	-	2	-	Possibly damaging	Neutral	Damaging
*EHMT1*	9,140638415,C,T	Exon6	c.1,043C > T	p.Ser348Leu	-	2	-	Possibly damaging	Deleterious	Damaging
*EHMT1*	9,140707493,A,G	Exon20	c.2,903A > G	p.Lys968Arg	ANK repeat domain	1	0	Possibly damaging	Neutral	Tolerated
*EHMT2*	6,31857330,C,-	Exon8	c.913_915delGGA	p.Glu305del	-	1	-	NA	NA	NA
*EHMT2*	6,31851617,G,A	Exon22	c.2,882C > T	p.Thr961Ile	SET domain	1	0	Possibly damaging	Neutral	Tolerated
*WIZ*	19,15535180,C,T	Exon7	c.2,039G > A	p.Arg680His	-	1	-	Probably damaging	Neutral	Damaging

Legend: ‘-’ denotes that the corresponding variant was not examined in control samples because it was located outside of a functional domain; ANK, ankyrin repeat domain; SET, Su(var)3-9-Enhancer of zeste-Trithorax domain.

Since histone methylation is effected through the formation of multimeric complexes of histone methyltransferases, which in turn are mediated by interaction of functional domains, we focused our interests on these regions. Results revealed that rare variants in the *EHMT1* ankyrin repeat domain (Lys968Arg) and *EHMT2* SET domain (Thr961Ile) were present in ASD cases but not in any of the 1,140 screened control subjects. Examining the cases, we observed no variations in the functional domains of *WIZ.* The case–control comparison showed no statistically significant association of any identified variants with ASD (Table 2). In addition, we also identified *EHMT1* and *EHMT2* variants that were present only in the control population (Additional file 4: Table S4).

**Table 2:** **Comparison of genotype and allele frequencies of** ***EHMT1*** **and** ***EHMT2*** **missense variants between autism spectrum disorder (ASD) cases and controls**

Gene	Variant	Subject	Genotype			***P***-value	Allele		***P***-value	MAF ^a^ (%)
*EHMT1*	c.2903A > G		A/A	A/G	G/G		A	G		G
	Lys968Arg	Autism	292	1	0	0.14	585	1	0.46	0.170
	(ANK repeat domain)	Control	1,139	0	0		2,278	0		0
*EHMT2*	c.2882C > T		C/C	C/T	T/T		C	T		T
	Thr961Ile	Autism	292	1	0	0.14	585	1	0.46	0.170
	(SET domain)	Control	1,139	0	0		2,278	0		0

^a^MAF: minor allele frequency. ANK, ankyrin repeat domain; SET, Su(var)3-9-Enhancer of zeste-Trithorax domain.

### Gene expression study

**Figure 3:**
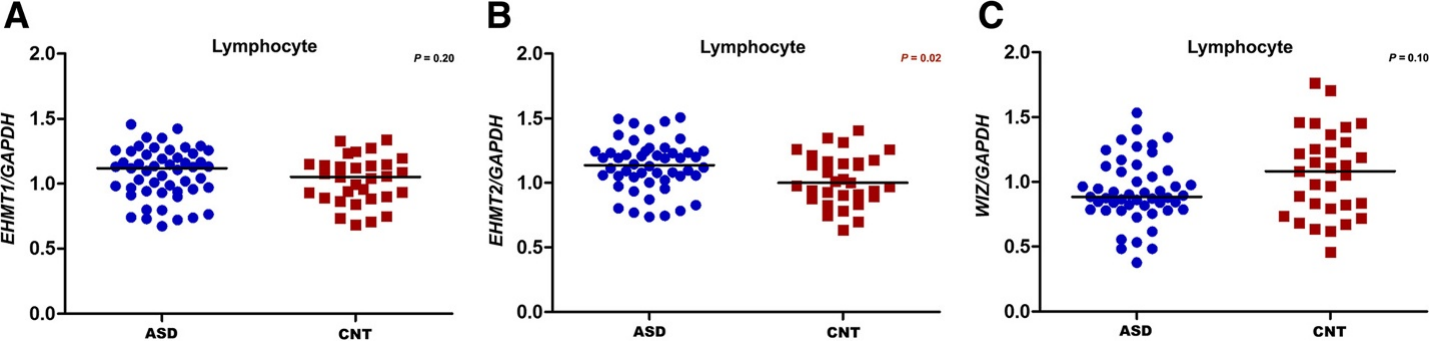
**Expression analysis of (A)** ***EHMT1,*** **(B)** ***EHMT2*** **and (C)** ***WIZ*** **in lymphocyte samples from a subset of autism spectrum disorder (ASD) cases and control (CNT) subjects who were resequenced for the candidate genes.**

**Figure 4:**
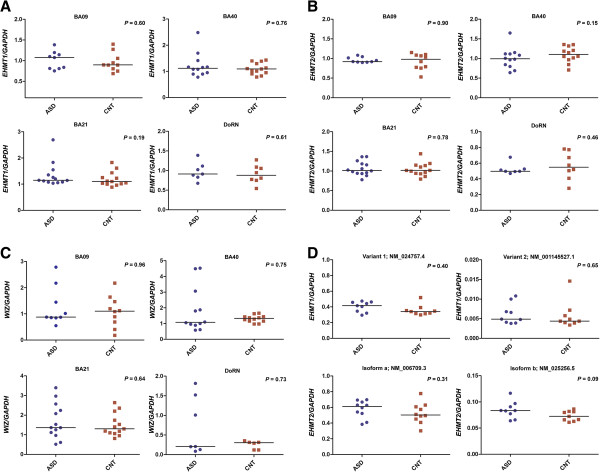
**Gene expression analysis of (A)** ***EHMT1,*** **(B)** ***EHMT2*** **and (C)** ***WIZ*** **in Brodmann area (BA) 09, BA21, BA40 and DoRN (dorsal raphe nucleus) of autism spectrum disorder (ASD) cases and controls (CNT). (D)** Isoform-specific expression analysis of *EHMT1* (variant 1: NM_024757.4 and variant 2: NM_001145527.1) and *EHMT2* (isoform a: NM_006709.3 and isoform b: NM_025256.5) in the prefrontal cortex (BA09) of ASD cases and controls.

The *EHMT2* transcript expression was significantly elevated in the peripheral blood cells of ASD when compared with control samples (*P* =0.02) (Figure 3B). But the *EHMT1* and *WIZ* levels were not significantly different between the ASD and control groups (Figure 3A, C). The gene expression analysis of *EHMT1, EHMT2* and *WIZ* in BA09, BA21, BA40 and DoRN regions from postmortem samples, showed no significant changes in expression levels between ASD and control groups (Figure 4A, B, C). We further examined the expression of *EHMT1* and *EHMT2* isoforms in the prefrontal cortex (BA09) of ASD patients. The *EHMT1* variant 1 (NM_024757.4) and *EHMT2* isoform a (NM_006709.3) were highly expressed compared to alternative isoforms. However, there was no significant difference in expression levels of these isoforms in the prefrontal cortex, when the ASD cases were compared to controls (Figure 4D).

## Discussion

Disruption of histone lysine methylation plays an important role in the pathogenesis of neurological disorders and cancer, as evidenced by the reports of genomic aberrations in histone methyltransferases in these diseases [10]. Since defective G9a and GLP histone lysine methyltransferases, give rise to autistic phenotypes [21], we searched for loss of function variants in the genes involved in H3K9 methylation, concentrating on rare mutations that show enrichment in ASD subjects. We focused on the variants located in the functional domains that are important in the formation of multimeric enzyme complex, and we identified the EHMT1 ankyrin repeat domain variant (Lys968Arg) and EHMT2 SET domain variant (Thr961Ile), which were present only in ASD cases and not in 1,140 control subjects. Although these two mutations were found exclusively in cases, case–control comparisons found no statistically significant association. Thus, our results did not support a role for these rare variants in ASD. This is in keeping with *in silico* analyses which predicted that the effects for both the *EHMT1* (Lys968Arg) and *EHMT2* (Thr961Ile) mutations would to be ‘neutral’ and ‘tolerated’ by Provean and SIFT, respectively, although PolyPhen2 predicted a ‘possibly damaging’ phenotype.

Since a large number of ‘loss of function’ variants are present in healthy human genomes [22], we speculate that the variants we identified may be private, owing to their lack of ‘predicted functional defects’, consistent through the three algorithms. On the other hand, balanced chromosomal abnormalities seen in ASD and related neurodevelopmental disorders are reported to disrupt the *EHMT1* gene [23]. In addition, a *de novo* deletion and rare inherited loss of function mutation in *EHMT1* were observed in a sporadic ASD trio sample [24] and in ASD families [25], respectively. It is clear that to understand the exact role of our identified variants, it will be necessary to examine them using much larger sample sets and more sophisticated functional assessments.

Interestingly, we observed an overexpression of the *EHMT2* gene in peripheral blood cells from ASD patients pointing towards a role of restricted chromatin state in ASD pathogenesis. A recent study showed increased expression of the *EHMT2* gene in lymphocytes and the *EHMT1* gene in both postmortem parietal cortex and lymphocyte samples, from patients with schizophrenia [26]. The study also found that a diagnosis of schizophrenia was a significant predictor for increased expression of histone methyltransferases. Therefore, the present results are interesting, given the genetic overlap between schizophrenia and ASD [27]. However, no significant changes in the expression levels of *EHMT1, EHMT2* or *WIZ* were observed in the postmortem brain samples from BA09, BA21, BA40 and DoRN region, between ASD subjects and controls. Additionally, we detected no differential expression of *EHMT1* and *EHMT2* isoforms in the prefrontal cortex (BA09) between the two subject groups. The results suggest an absence of common variants in the regulatory genomic elements of these genes associated with ASD.

Mutations in the chromatin remodeling enzymes have been reported in psychiatric diseases, which disrupt the chromatin regulation leading to altered neuronal function and behavioral abnormalities [28]. But in our study, such a loss of function mutation was not observed. Moreover, the identified mutations did not have a cogent effect in ASD pathogenesis, either through functional deficits or changes in expression levels. Therefore, it can be concluded that the loss of function mutations in histone methyltransferases may constitute a rare event in ASD pathogenesis, which is supported by the fact that H3K9 modifying enzymes have fewer reported mutations, when compared to other chromatin regulators [29].

Since *EHMT2* overexpression correlates with the increased H3K9me2 levels [30], it could result in the repressed transcription of the genes/genetic network relevant to ASD pathogenesis. However, the results from expression analysis of peripheral blood cells should be interpreted cautiously because peripheral blood chromatin may not essentially provide information specific to a brain region or neuronal phenotype [31]. Future studies are warranted to profile the global H3K9 (mono and di) methylation status in ASD brain to delineate the genetic networks, which are dysregulated in ASD.

Although the present study did not show statistically significant enrichment of variants in ASD, their possible contribution to disease cannot be ruled out, due to the relatively small sample size restricting the statistical power of this study and also the absence of identified patient-specific mutations in global databases for the control population. From the available three-dimensional structures, it would appear that both mutations are located on the surface of the proteins (Additional file 3: Figure S1 (B and C), implying a potential role for the variants in complex formation. Recent whole genome and exome sequencing studies have clearly shown a heterogeneous genetic basis for ASD and have identified a large number of candidate genes, converging on functional pathways of neuronal signaling and development, synapse function and chromatin regulation [32]. It is also known that SETDB1 and Suv39h1 co-exist in the H3K9 methylation multimeric complex, with interdependent functionality [33]. Therefore, the polygenic burden of ASD may mask the effects of single rare variants, obscuring their individual contribution to disease pathogenesis [34].

## Conclusion

In summary, we identified two novel, rare missense variants in the *EHMT1* and *EHMT2* genes from ASD patients. We surmise that these variants alone may not be sufficient to exert a significant effect on ASD pathogenesis and that a concerted interaction with additional genetic or epigenetic effects may be needed to manifest the disease phenotype. The elevated expression of *EHMT2* observed in peripheral blood cells from ASD patients may support the notion of a restrictive chromatin state in ASD pathogenesis, similar to schizophrenia. Future studies with larger sample sizes and sophisticated functional assessments are warranted to define the precise role of *EHMT1* and *EHMT2* in ASD pathogenesis.

## Authors’ information

Kayoko Esaki: Research Fellow of Japan Society for the Promotion of Science.

## Electronic supplementary material

Additional file 1: Table S1: Demographic details of autism spectrum disorder (ASD) and control brain samples from the NICHD Brain and Tissue Bank, University of Maryland School of Medicine (http://medschool.umaryland.edu/btbank/). (DOCX 16 KB)

Additional file 2: Table S2: PCR amplification primers and conditions. (XLSX 19 KB)

Additional file 3: Figure S1: (A) Domain structure of EHMT1 (GLP) and EHMT2 (G9a), indicating mutated and their conserved positions, (B) three-dimensional structure of EHMT1 (GLP), and (C) three-dimensional structure of EHMT2 (G9a). The structural data were obtained from Protein Data Bank (http://www.rcsb.org/pdb/home/home.do) and visualized using the UCSF Chimera package (http://www.cgl.ucsf.edu/chimera/) for determining the position of identified variants. The EHMT1/GLP complex (PDB entry: 3B95) contains three peptide chains, where the A and B chains are from GLP, and the P chain is a histone H3 N-terminal peptide. The B chain (blue), P chain (green) and the variant (red) are shown in figure (B). The mutation is located on the surface of the protein. The EHMT2/G9a complex (PDB entry: 3K5K) contains two SET domains from G9a (A and B chains). The A chain is shown here in (C) with ligands DXQ (7-[3-(dimethylamino) propoxy]-6-methoxy-2- (4-methyl-1,4-diazepan-1-yl)-N-(1-methylpiperidin-4-yl)quinazolin-4-amine) and S-adenosyl-L-homocysteine marked in green and cyan, respectively. The variant position (red) is located on the surface of the protein, away from substrate binding sites. (XLSX 9 KB)

Additional file 4: Table S3: List of TaqMan assay IDs used for gene expression studies. (XLSX 17 KB)

Additional file 5: Table S4: Novel and previously reported variants in the ASD cohort and variants specific to the control population. (PDF 2 MB)

## Abbreviations

ADI-R: Autism Diagnostic Interview-Revised
ASD: autism spectrum disorders
BA: Brodmann’s area
CNT: control
DoRN: dorsal raphe nucleus
DSM-IV-TR: *Diagnostic and Statistical Manual, Fourth Edition, Text Revision*
MAF: minor allele frequency
NICHD: National Institute of Child Health and Human Development
RT-PCR: reverse transcription polymerase chain reaction
SCID: Structured Clinical Interview for DSM-IV
SET: Su(var)3-9-Enhancer of zeste-Trithorax domain.
