# Supplementary material for: Exon resequencing of H3K9 methyltransferase complex genes, EHMT1, EHTM2 and WIZ, in Japanese autism subjects
Source: Mol Autism. 2014 Oct 6;5:49. doi: 10.1186/2040-2392-5-49 (PMC4233047; doi:10.1186/2040-2392-5-49)
Supplement: Supplementary file 1 — Additional file 1: Table S1: Demographic details of autism spectrum disorder (ASD) and control brain samples from the NICHD Brain and Tissue Bank, University of Maryland School of Medicine (http://medschool.umaryland.edu/btbank/). (DOCX 16 KB) [file 13229_2014_145_MOESM1_ESM.docx]

**Additional_file_1 Table S1**: Demographic details of ASD and control brain samples from the NICHD Brain and Tissue Bank, University of Maryland School of Medicine (<http://medschool.umaryland.edu/btbank/>)

| **Brain Region** | **Age (Mean ± SD)** | | **Gender** | | **PMI* (Mean ± SD)** | | **pH (Mean ± SD)** | | **RIN^#^ (Mean ± SD)** | |
| --- | --- | --- | --- | --- | --- | --- | --- | --- | --- | --- |
|  | **Autism** | **Control** | **Autism** | **Control** | **Autism** | **Control** | **Autism** | **Control** | **Autism** | **Control** |
| BA09 | 13.50 ± 5.87 | 13.70 ± 5.71 | 3 F , 7 M | 3 F , 7 M | 22.50 ± 12.86 | 16.60 ± 7.32 | 6.38 ± 0.50 | 5.96 ± 0.40 | 5.75 ± 1.39 | 6.29 ± 1.02 |
| BA21 | 12.21 ± 5.61 | 12.42 ± 5.44 | 4 F , 10 M | 4 F , 10 M | 22.35 ± 12.36 | 16.42 ± 6.29 | 6.42 ± 0.44 | 6.03 ± 0.34 | 4.84 ± 1.58 | 5.79 ± 1.28 |
| BA40 | 12.21 ± 5.61 | 12.84 ± 5.42 | 4 F , 10 M | 4 F , 9 M | 22.35 ± 12.36 | 16.76 ± 6.41 | 6.42 ± 0.44 | 6.03 ± 0.34 | 5.84 ± 1.57 | 6.03 ± 0.96 |
| DoRN | 15.50 ± 4.56 | 15.75 ± 4.16 | 3 F , 5 M | 2 F , 6 M | 23.00 ± 14.54 | 14.62 ± 8.05 | 6.24 ± 0.44 | 6.04 ± 0.40 | 3.41 ± 0.64 | 4.38 ± 0.95 |

* Post mortem interval; RIN, ^#^RNA Integrity Number; F, Female; M, Male
