# Supplementary figures and images for: Exon resequencing of H3K9 methyltransferase complex genes, EHMT1, EHTM2 and WIZ, in Japanese autism subjects
Source: Mol Autism. 2014 Oct 6;5:49. doi: 10.1186/2040-2392-5-49 (PMC4233047; doi:10.1186/2040-2392-5-49)

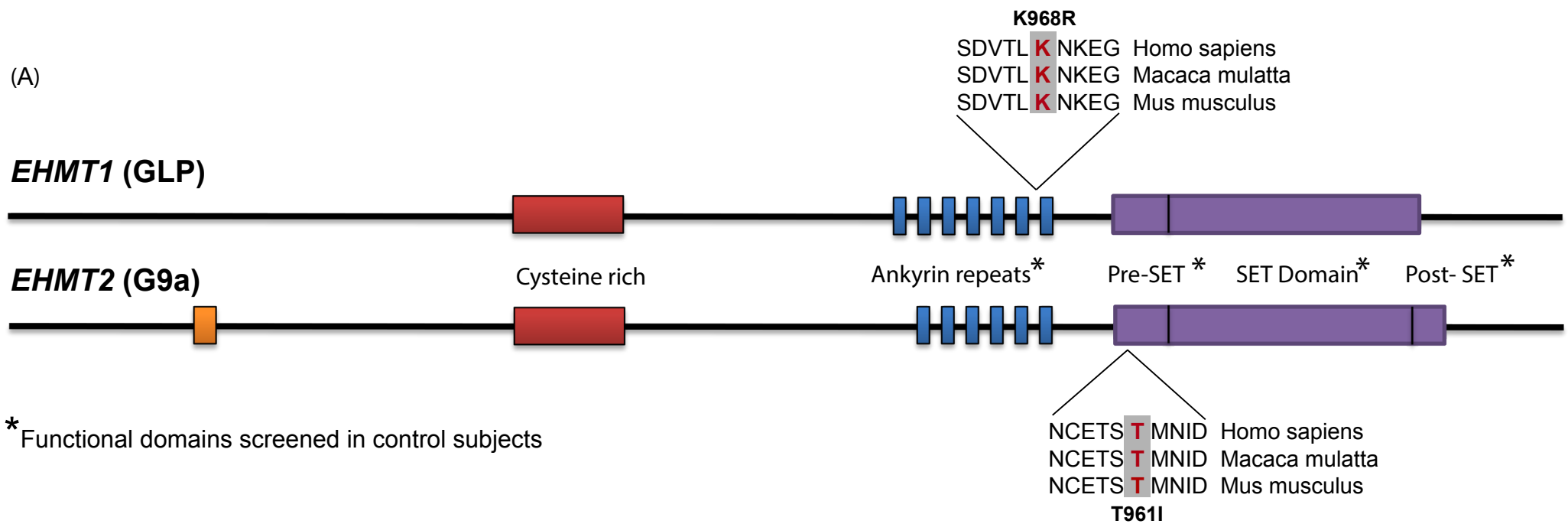

(B)

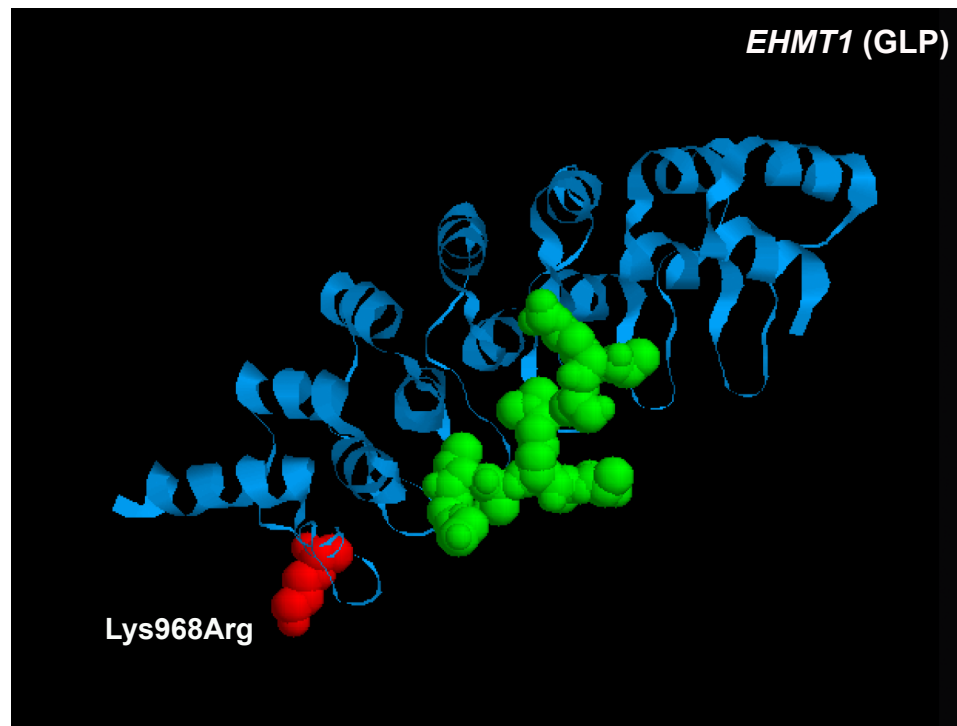

(C)

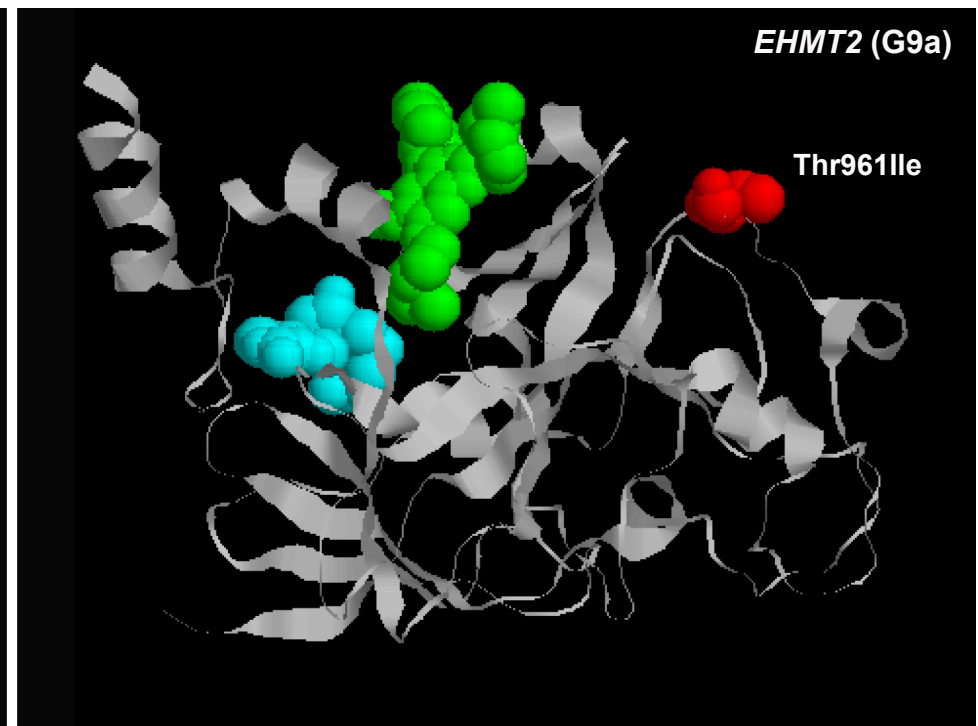

Supplement: Supplementary file 5 — Additional file 5: Table S4: Novel and previously reported variants in the ASD cohort and variants specific to the control population. (PDF 2 MB) [file 13229_2014_145_MOESM5_ESM.pdf]
